# Cardiovascular Inflammation

**DOI:** 10.1155/2013/123513

**Published:** 2013-07-31

**Authors:** Miao Wang, Weijun Jin, Austin Meng Guo, Jane Stubbe

**Affiliations:** ^1^State Key Laboratory of Cardiovascular Disease, Clinical Pharmacology Center, Fuwai Hospital, National Center for Cardiovascular Diseases, Chinese Academy of Medical Sciences and Peking Union Medical College, 12th Floor, Keyanlou Building, 167 Beilishi Road, Xicheng District, Beijing 100037, China; ^2^Department of Cell Biology, SUNY Downstate Medical Center, BSB, 2-106, 450 Clarkson Avenue, Brooklyn, NY 11203, USA; ^3^Department of Pharmacology, New York Medical College, 15 Dana Road, Room 546B, Valhalla, NY 10595, USA; ^4^Institute of Molecular Medicine, Department of Cardiovascular and Renal Research, University of Southern Denmark, J. B. Winsloewvej 21, 3rd floor, 5000 Odense, Denmark

Atherosclerotic cardiovascular disease (AVD) is the leading cause of death and morbidity in the western world. Since first proposed in 1999 by Ross, inflammation has been gradually accepted to be one of the key components in development of atherosclerosis and in its acute clinical manifestations such as heart attacks and strokes [1]. This special issue includes a series of papers that cover a diverse spectrum of this topic. It is aimed to provide the readers a concurrent understanding of the fundamental development as well as molecular insights of AVD thus facilitating therapeutic discovery by targeting cardiovascular inflammation. 

First, the decade-old concept that atherosclerosis is a chronic inflammatory disease is reinforced by a clinical observation published in this issue by Su et al. that serum IL-6, a hallmark of inflammation, is significantly associated with all-cause- and cardiovascular mortality in hospitalized ethnic Han Chinese patients with coronary artery disease. 

The study reveals the positive correlation between serum IL-6 levels and these events. Interestingly, another recent study demonstrates that serum IL-6 concentrations are associated with mortality in elderly adults [2]. 

Second, from disease pathogenesis perspective, endothelium activation by physiological stimulation, such as sheer stress, or by inflammatory insults, is an early event of atherogenesis and may also contribute to late thrombotic event [3]. Krötz et al. investigated the role of endothelial tyrosine phosphatase (SHP-1) in platelet-endothelium interaction and arterial thrombosis. SHP-1 is known to negatively regulate endothelial superoxide production [4]. They showed that SHP-1 likely serves as an autoinhibitory molecule to prevent excess inflammatory response and thrombus formation, which is crucial in TNF*α*-induced endothelial inflammation.

Following endothelial activation, leukocytes and monocytes in particular are recruited to the artery wall where they initiate local inflammation, thereby underscoring early atherogenesis [5]. Binding of chemokines to their G-protein-coupled receptors (GPCRs) is essential for monocyte migration and activation [6]. Patel et al. reviewed the regulation of chemokine receptor signaling in the context of atherosclerosis. They proposed that targeting the molecules in the GPCR signaling pathway, such as GPCR kinases (GRKs), arrestin proteins, and regulator of G-protein signaling (RGS) proteins, may limit cardiovascular inflammation and provide new strategies of therapeutic discovery for atherosclerosis. Leukocyte trafficking to the inflammatory site is highly regulated in infection. The involved mechanisms may also underlie cardiovascular inflammation. Using a mouse model of peritoneal inflammation, Lam et al. shows that reversible chemotactic gradients between peritoneal exudates and blood exist in both basal and inflamed conditions, which may play a role in directing leukocyte trafficking.

As atherosclerotic lesion advances, vascular smooth muscle cells and leukocytes release paracrine substances that affect the extracellular matrix (ECM) composition [7]. In particular, degradation of the ECM weakens the plaque eventually resulting in plaque rupture, which is likely to cause acute events such as stokes and acute myocardial infarctions [8]. Elevated levels of matrix metalloproteinases (MMPs) in plaques are suspected to be a major underlying cause of plaque rupture [9]. The gelatinases MMP-2 and MMP-9 have been mainly in focus [10]. The recent contribution of MMP-8, which initially was believed to be restricted to neutrophils but now is known to be associated with multiple cell types within the plaque [11], is reviewed in this special issue by Lenglet et al. They display the involvement of MMP-8 in the remodeling processes within the atherosclerotic plaque that promotes its rupture. Furthermore, accumulating evidence associates MMP-8 gene variation with atherogenesis. The authors suggest that new studies need to be designed to evaluate the role of MMP-8 as a potential biomarker, which could be a part of the multipanel biomarker system in predicting cardiovascular events. 

Pentraxin 3 (PTX3) is an acute phase protein-like C-reactive protein (CRP). It belongs to the pattern recognition family and is involved in the complement-mediated clearance of apoptotic cells [12, 13]. Bonacina et al. thoroughly reviewed the role of PTX3 in cardiovascular inflammation. Although the majority of data favor a physiologically protective role of PTX3 against cardiovascular inflammation, its exact role and therapeutic value await further examination.

Finally, noninvasive imaging tools have been increasingly used to assess cardiovascular disease. They are particularly informative by allowing longitudinally assessment of cardiovascular disease progress as well as functional/structure changes. Escher et al. demonstrated that speckle tracking echocardiography is a useful adjunctive assisting tool for evaluation over the course of intramyocardial inflammation in patients with acute myocarditis and chronic cardiomyopathy, although an unequivocally confirmed diagnosis by endomyocardial biopsies is still required. 

Collectively, this special issue aims at providing insight into some key aspects of cardiovascular inflammation, to which new therapeutic discovery may be directed.

*Miao Wang* *Miao Wang* *Weijun Jin* *Weijun Jin* *Austin Meng Guo* *Austin Meng Guo* *Jane Stubbe* *Jane Stubbe*
